# Preliminary Pilot-Testing of Social Determinants of Health Screener for Individuals With Intellectual and Developmental Disabilities in Med-Peds

**DOI:** 10.7759/cureus.38541

**Published:** 2023-05-04

**Authors:** Emily Hotez, Kristine J Chua, Nathan Samras, Andrew M Smith, Alice Kuo

**Affiliations:** 1 General Internal Medicine, University of California Los Angeles, David Geffen School of Medicine, Los Angeles, USA

**Keywords:** intellectual and developmental disabilities, interdisciplinary health team, social determinants of health (sdoh), screening, med peds

## Abstract

In the United States, one in six children has an intellectual and/or developmental disability (I/DD), including attention deficit hyperactivity disorder (ADHD), autism, cerebral palsy, learning disabilities, seizures, and developmental delays, with or without intellectual impairment. Individuals with I/DDs experience disproportionate rates of immune, metabolic, cardiovascular, and neurological disorders, as well as anxiety, depression, functional somatic symptoms, and other co-occurring physical and mental health conditions. During the coronavirus disease 2019 (COVID-19) pandemic, having an I/DD emerged as one of the strongest predictors of contracting and dying from COVID-19. These findings spurred increased attention toward the myriad health inequities affecting this population well before the pandemic. While inequities for individuals with I/DD can be traced to many factors, social determinants of health (SDOH) - the underlying social, economic, and environmental conditions that lead to poor health outcomes and high healthcare costs - are key contributors. Our interdisciplinary combined internal medicine and pediatrics (Med-Peds) team of physicians, psychologists, and researchers within a large, diverse, academic health system aimed to pilot-test the implementation of a five-item SDOH screener within a Med-Peds specialty clinic focused on the developmental needs of individuals with I/DD and their families (Leadership Education in Neurodevelopmental Disabilities {LEND}) and a general primary care practice (PCP). The SDOH screener tested in this initiative includes five items from the Accountable Health Communities (AHC) Health-Related Social Needs Screening Tool (HRSN) assessing social isolation, food insecurity, transportation, and paying for basic needs, such as housing and medical care. In this study, we describe the process of implementing this screener and collecting initial pilot data from 747 patients between October 2022 and April 2023 across the LEND and the primary care practice. We also highlight the challenges and opportunities identified during the mid-way point of implementation and pilot testing. The results of this pilot study revealed low response rates among SDOH screeners, spurring several measures to increase uptake, including increasing the accessibility of the screener and ensuring the screener results in effective referrals. We call on additional Med-Peds healthcare teams without universal SDOH screening protocols in place - particularly those serving the I/DD population - to consider adopting these practices.

## Introduction

During the COVID-19 pandemic, having an intellectual and/or developmental disability (I/DD), including autism, attention deficit hyperactivity disorder (ADHD), cerebral palsy, and learning disabilities, emerged as one of the strongest predictors of contracting and dying from coronavirus disease 2019 (COVID-19) [1-3]. These findings spurred increased attention toward the myriad health inequities affecting this population well before the pandemic, including disproportionate rates of immune, metabolic, cardiovascular, and neurological disorders, as well as anxiety, depression, functional somatic symptoms, and other co-occurring physical and mental health conditions [4,5]. While inequities for individuals with I/DD can be traced to many factors, social determinants of health (SDOH) - the underlying social, economic, and environmental conditions that lead to poor health outcomes and high healthcare costs - are key contributors [6].

SDOH affect an estimated 80% of health outcomes in the United States general population [7]. SDOH are particularly salient for individuals with I/DD, who not only experience distinct socioeconomic burdens [8], but also pronounced psychosocial challenges (social isolation, exclusion, and discrimination), experiences of abuse and trauma, and lifelong struggles accessing quality healthcare [9-15].

SDOH screening and referral in primary care have been found to increase receipt of social services among patients with I/DD [16]. This research, however, finds that screening activities may decline after initial training and education surrounding utilizing the screener [16]. Indeed, healthcare providers serving individuals with I/DD as well as the general population are challenged in screening for SDOH-related issues experienced by their patients, which impedes their capacity to connect patients with health-promoting services, supports, and resources [17,18]. As a result, the healthcare system is limited in its capacity to proactively identify and address important upstream mechanisms potentially perpetuating health inequities for the I/DD population [17,18].

Combined internal medicine and pediatrics (Med-Peds) healthcare providers are uniquely positioned to spearhead SDOH screening and referral efforts due to their role in supporting patients in the transition from pediatric to adult care and thereafter [19]. Med-Peds providers are crucial facilitators of effective SDOH screening and referral practices for all patients, but particularly for individuals with I/DD and their families who disproportionately experience healthcare fragmentation between the pediatric and adult healthcare systems [20-23].

Our interdisciplinary Med-Peds team of physicians, psychologists, researchers, data analysts, and IT professionals within a large, diverse, academic health system aimed to pilot-test the implementation of a five-item SDOH screener within a Med-Peds specialty clinic focused on the developmental needs of individuals with I/DD and their families. As part of this same initiative and to inform future efforts to scale our initiative, we also identified the utility of implementing this screener in a general primary care practice (PCP). The SDOH screener tested in this initiative includes five items from the Accountable Health Communities (AHC) Health-Related Social Needs Screening Tool (HRSN) assessing social isolation, food insecurity, transportation, and paying for basic needs such as housing and medical care. In this study, we describe a workflow process initiated within the specialty clinic and expanded to the general primary care practice. The overarching goal of this manuscript is to highlight the challenges and opportunities identified during the mid-way point of implementation and pilot testing.

We anticipate that this initiative will have important impacts on the field, including spurring the development of learning networks, comprised of providers and clinics interested in initiating SDOH screening and referral efforts in their clinics. This will, in turn, increase the extent to which the United States healthcare system is responsive to the social needs and resulting health inequities in marginalized populations.

## Materials and methods

Clinic overview

The current project focal clinic, currently operating entirely via telemedicine, adheres to the Leadership Education in Neurodevelopmental Disabilities (LEND) model and is one of the 60 LEND clinics in the United States [24]. The LEND model seeks to promote high-quality primary and psychological care and coordinate services across the life course for individuals with I/DD [24,25].

To test whether our process in LEND would be acceptable, feasible, and relevant to other clinics, potentially those who serve both the general and I/DD populations (i.e., primary care practices), we have begun to replicate this process in a larger primary care clinic. Both clinics are situated within a Med-Peds department in a large clinic network; the primary care clinic, however, is more inclusive of the general patient population and does not specifically focus on the I/DD population. The patient population includes a range of patients across the typical spectrum of primary care, from pediatrics through geriatrics.

Current data capacity within both clinics is limited; efforts are underway to improve the collection and tracking of demographic data as part of this initiative. As an example, the leadership team (described in detail below) is working with healthcare providers within the target clinics, as well as leadership within the larger healthcare system, to understand the challenges and opportunities of systematic data collection of race, ethnicity, and other key indicators. The team is also consulting with data and IT professionals in building a dashboard that would facilitate seamless extraction and analysis of these indicators. There are two reliable demographic indicators (age and sex assigned at birth) available in the data (reported for our sample in the Results section). Both clinics, however, are located within Los Angeles County, which is comprised of a significant Hispanic population (49.1%) as well as a significant proportion of individuals living in poverty (14.1%) [26].

Procedure

**Figure 1:**
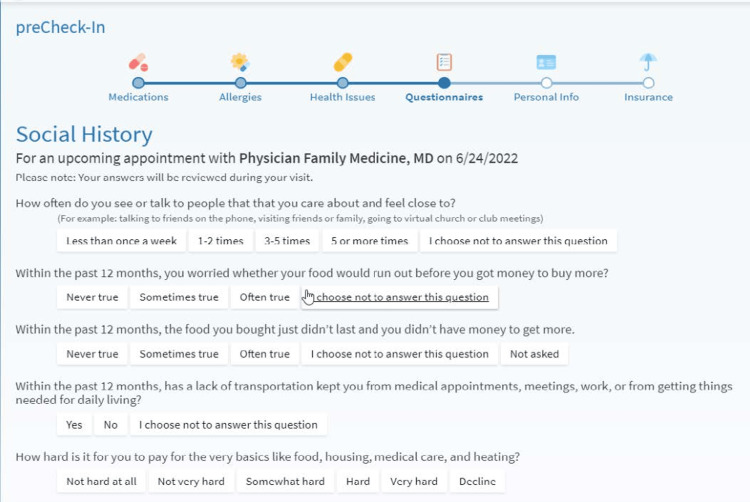
Social determinants of health (SDOH) screener.

In the current project, our pilot test focused on developing and implementing an automated process for electronic release of a five-item SDOH screener to all patients with visits in the project time frame (between October 2022 and April 2023), prior to their visit (Figure 1). The SDOH screener tested in this initiative includes five items from the Accountable Health Communities (AHC) Health-Related Social Needs Screening Tool (HRSN) assessing social isolation, food insecurity, transportation, and paying for basic needs, such as housing and medical care. This measure was selected based on its utilization by select clinics in the larger health system. Consistent with previous efforts to initiate SDOH screening in healthcare systems [27], as well as principles of effective quality improvement (QI) [28,29], we utilized a step-wise and iterative approach. This approach consists of three phases.

The first step was to formulate an interdisciplinary team comprised of LEND practitioners and administrators - led by the clinic director, who serves as a physician “champion” for the initiative - as well as a leadership team comprised of Med-Peds physicians with QI and SDOH expertise and researchers with evaluation expertise. Receipt of funding for clinic-wide QI project facilitated these cross-discipline collaborations. This first step took place over a six-month period (November 2021-April 2022).

The second step was to engage in planning and workflow mapping. This process involved collaborating with an electronic medical records (EMR) system analytic team, comprised of data analysts and IT professionals, to build the SDOH questionnaire and automatically release it to eligible patients prior to their visit. The leadership team in consultation with the EMR analytic team offered periodic training to LEND clinic staff to ensure back-end operations were disseminating the screener effectively.

This process also entailed making critical decisions that would affect implementation. As an example, given the expansive age range of Med-Peds patients, an important decision involved the age at which a patient, as opposed to a caregiver or supporter, would complete the screener. We relied on previous research that finds that autistic children’s self-reported data aligns with caregiver-reported data as early as age 10 years, as well as clinical insight, to determine that patients aged 12 years and over could feasibly complete the screener themselves [30].

Additional feedback from the LEND team was continuously solicited to increase feasibility and workflow efficiency. Overall, the activities occurring within this second step of the process took place over an approximately six-month period (May 2022-October 2022), which was longer than initially anticipated. The time-intensive nature of this step was due to delays and logistical challenges with building data processes and protocols in the midst of many competing requests received by data analysts and IT professionals within the health system.

**Figure 2:**
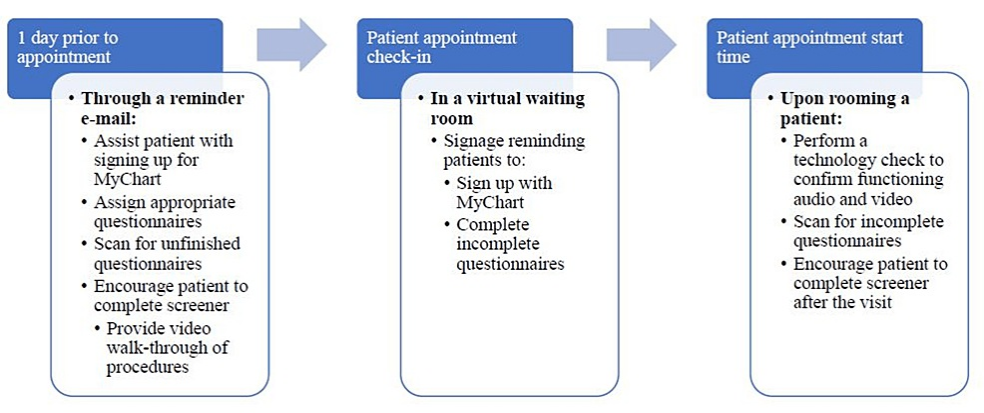
Social determinants of health (SDOH) screening workflow. MyChart (Verona, WI: Epic Systems Corporation)

The third step-currently in progress-entails an initial implementation process (October 2022 to present). Implementation involved integrating the SDOH screening process into workflow practices and identifying steps toward maximizing effectiveness. The goal implementation process we are working towards is depicted in Figure 2. Presently, the SDOH screener is auto-released to eligible patients prior to each new patient visit via a reminder e-mail. The patient completes the SDOH screener prior to the start of the physician-patient interaction and responses are automatically entered into the EMR. The video walk-through and virtual waiting room are not currently implemented and are described as target next steps in the Results section.

**Figure 3:**
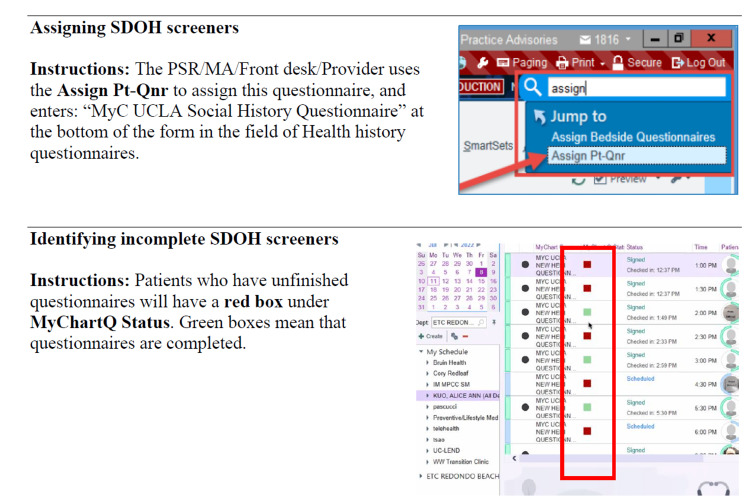
Provider tipsheet examples. SODH: social determinants of health; PSR/MA: patient services representative/medical assistant; Pt-Qnr: patient questionnaire; MyC: MyChart; UCLA: University of California, Los Angeles

The leadership team created a provider “tip sheet,” complete with electronic record screenshots to further support providers in ensuring patients complete the screener. Providers can refer to the tip sheet for ongoing consultation and guidance (Figure 3).

We were able to begin implementation in the primary care clinic soon after implementation commenced in the LEND clinic (October 2022), given the extensive infrastructure-building process prior to implementation. Between October 2022 and April 2023, 94 SDOH screeners have been administered in LEND; 2,522 SDOH screeners have been administered in the general PCP.

## Results

Table 1 below displays the results of this study. Between October 2022 and April 2023, 94 SDOH screeners have been administered in LEND. Of those screeners, only nine were completed, yielding a 10% response rate (female: n=4; male: n=9; mean age=15.4). Within the completed screeners, five screens were positive and triggered referrals to community-based organizations. Also in this time frame, within our general primary care clinic, 2,522 SDOH screeners have been administered. Of those screeners, 738 have been completed, yielding a 43% response rate (female: n=453; male: n=238; mean age=37.72). Within the completed screeners, 143 screens were positive and triggered referrals to community-based organizations.

**Table 1:** SDOH screeners administered/completed and positive screens. SDOH: social determinants of health; LEND: Leadership Education in Neurodevelopmental Disabilities; PCP: primary care practice

Clinic	SDOH screeners administered, October 2022-April 2023 (n)	SDOH screeners completed (n, % of administered)	SDOH positive screens: 1+ SDOH need identified (n, % of completed)
LEND	93	9, 10%	5, 56%
PCP	2,522	738, 43%	143, 19%

Given the low uptake of the SDOH screener, particularly in LEND, other results of this pilot test included the generation of next steps to maximize the uptake among the I/DD population that may also be beneficial for the general population. First, we are currently optimizing screener accessibility by creating a complementary video walk-through and a customized virtual waiting room. The video will provide instructions to patients utilizing multiple modalities (e.g., visual and oral cues via clear narration, captioning, and graphics), in alignment with best practices in accessibility [31]. The video walk-through will be integrated into the reminder e-mail sent to patients prior to their appointments.

Second, we are creating a customized virtual waiting room that will provide clear virtual signage and instructions for accessing and completing the screener. If the patient has not completed the SDOH screener prior to the visit, they will receive an additional reminder to complete the screener upon entering the virtual waiting room.

Finally, we are soliciting feedback from all LEND clinicians about the services, supports, and resources they refer patients to following positive SDOH screens. This feedback will inform the refinement and enhancement of the existing SDOH referral list for the specific needs of the patient population. The above considerations will also be applied to the general primary care practice and will allow for minimal burden and maximal expediency for clinic staff, as well as improve the effectiveness of the SDOH screening and referral effort.

## Discussion

Initiating SDOH screening efforts in Med-Peds clinics is crucial for ensuring that patients have access to SDOH-related services, supports, and resources across the life course. SDOH are particularly salient for individuals with I/DD, who not only experience distinct socioeconomic burden [8], but also pronounced psychosocial challenges; social isolation, exclusion, and discrimination; experiences of abuse and trauma; and lifelong struggles accessing quality healthcare [9-15]. The current pilot study presents preliminary insights from initiating SDOH screening and referral efforts within both specialty and general Med-Peds clinics. The presentation of this pilot study can inform national efforts to implement, test, and scale universal SDOH screening and referral practices in Med-Peds.

Beyond spurring the development and implementation of supplemental efforts to improve access and uptake of the screeners among patients with specific support needs (described in Results section), our efforts yielded several insights. First, our efforts underscore the importance of cultivating an interdisciplinary team in SDOH screening and referral efforts. Our team was purposefully formed to be inclusive of clinicians with patient-related expertise and access, researchers with the capacity to conduct ongoing evaluation of processes and outcomes, and data analysts to enhance clinic capacities to collect and analyze data. Med-Peds healthcare providers who treat patients across the life course with diverse needs, particularly those who are based in health systems with access to research and data personnel, may be uniquely positioned to spearhead the interdisciplinary efforts required for this work [19-23].

Second, this work highlighted the importance of utilizing QI methodologies that are conducive to a process that is continuously and iteratively revised. This insight aligns with previous efforts that emphasize the utility of this approach [28,29]. The capacity to adjust procedures, identify needed resources, and make data-informed decisions and pivots ensure that screening and referral processes are feasible, acceptable, and effective for the clinic. Indeed, our QI approach allowed us to identify modifications and accommodations that may be helpful in improving screener uptake within the I/DD population and potential for the general population.

Finally, the insights gleaned from this project shed light on the potential challenges that clinics across the country encounter in their efforts to identify and respond to SDOH-related needs, experiences, and priorities among their patients. These challenges are, in part, documented in the literature, but may be compounded by logistical difficulties often only uncovered through QI projects [17,18]. Moving forward, it will be particularly fruitful for clinics to forge multi-site collaborations and learning networks focused on SDOH screening and referral.

The current effort is not without its limitations. To be sure, implementing SDOH screening and referral practices within individual specialty clinics is important and may be advantageous in ensuring that these efforts meet the specific needs of particular patient populations. This approach can also aid in demonstrating feasibility and acceptability within larger health systems without universal SDOH screening and referral practices. This approach, however, may not represent the most scalable option for promoting universal screening in the long term. Moving forward, our most significant hurdles relate to promoting effective wide-scale SDOH screener adoption within our larger health system and potentially across the 60 LEND clinics nationwide. Successful sustainability and scalability will be contingent on our capacity to continuously collect, interpret, and disseminate data collected as part of this project, making our researcher-provider partnerships a critical aspect of our efforts moving forward.

## Conclusions

This pilot study presents preliminary insights from a quality improvement (QI) project promoting SDOH screening and referral efforts within both specialty and general Med-Peds clinics. Throughout these efforts, we prioritized the implementation and evaluation of QI efforts for patients with intellectual and/or developmental disabilities (I/DD) due to their pronounced SDOH-related needs identified in the literature.

We anticipate that the presentation of this pilot study, as well as our ongoing challenges and planned next steps, can inform national efforts to implement, test, and scale universal SDOH screening and referral practices in Med-Peds clinics. We call on additional Med-Peds healthcare teams without universal SDOH screening protocols in place, particularly those serving the I/DD population, to consider adopting these practices and build a learning network to bolster their effectiveness.
